# Comparing Outcomes of Two Antiviral Therapy Combinations among COVID-19 Patients

**DOI:** 10.1155/2022/1522426

**Published:** 2022-01-07

**Authors:** Hossein Mazaherpour, Masoomeh Sofian, Elham Farahani, Alireza Abdi, Sakine Mazaherpour, Anahita Bavand, Amitis Ramezani

**Affiliations:** ^1^Arak University of Medical Sciences, Arak, Iran; ^2^Infectious Diseases Research Center, Arak University of Medical Sciences, Arak, Iran; ^3^Kermanshah University of Medical Sciences, Kermanshah, Iran; ^4^Treatment Management of Social Security Organization of Khuzestan Province, Mahshahr, Iran; ^5^Clinical Research Department, Pasteur Institute of Iran, Tehran, Iran

## Abstract

Several therapeutic regimens for COVID-19 have been studied, such as combination antiviral therapies. We aimed to compare outcome of two types of combination therapies atazanavir/ritonavir (ATV/r) or lopinavir/ritonavir (LPV/r) plus hydroxychloroquine among COVID-19 patients. 108 patients with moderate and severe forms of COVID-19 were divided into two groups (each group 54 patients). One group received ATV/r plus hydroxychloroquine, and the other group received hydroxychloroquine plus LPV/r. Then, both groups were evaluated and compared for clinical symptoms, recovery rates, and complications of treatment regimens. Our findings showed a significant increase in bilirubin in ATV/r-receiving group compared to LPV/r receivers. There was also a significant increase in arrhythmias in the LPV/r group compared to the ATV/r group during treatment. Other findings including length of hospital stay, outcome, and treatment complications were not statistically significant. There is no significant difference between protease inhibitor drugs including ATV/r and LPV/r in the treatment of COVID-19 regarding clinical outcomes. However, some side effects such as hyperbilirubinemia and arrhythmia were significantly different by application of atazanavir or lopinavir.

## 1. Introduction

Coronavirus Disease 2019 (COVID-19) started in late 2019 in Wuhan, China, which then spread rapidly to many countries worldwide [1, 2]. This pandemic disease has infected more than 200 million people worldwide [2, 3] caused by a single-stranded RNA virus with human and animal hosts [4–6]. The average incubation period of the disease is between 4 and 5 days, but it may last up to 14 days. Some data show that SARS-CoV-2 with mild presentation leads to short protection rather than severe infection [5, 7–9]. The disease may differ from a mild and asymptomatic form to a severe presentation with acute respiratory distress syndrome (ARDS) and death. According to the results of the studies, about 81% of patients presented moderate and mild forms whereas 14% and 5% had severe and threatening to fatal forms of the disease, respectively [10–12]. Symptoms including fever, cough, and dyspnea have been found in about 70% whereas muscle pain and headache were seen in 36% and 34%, respectively [13].

Several therapeutics and vaccines have been investigated to overcome COVID-19 disease [6, 14]. Among these drugs, protease inhibitors which are used for treatment of HIV-1 are applied in COVID-19 [15]. Protease inhibitors impair the virus replication by inhibition of protease, which are hepatically metabolized via the CYP isoenzyme CYP3A4. Lopinavir/ritonavir (LPV/r) is a potent inhibitor of CYP3A4 metabolism; therefore, it induces more drug interactions [16]. Moreover, LPV/r, as a protease inhibitor drug, can inhibit the protease 3CLpro enzyme, which is one of the RNA polymerase-dependent proteases of the virus and is involved in virus replication [15]. Atazanavir/ritonavir (ATV/r) is another drug belonging to the protease inhibitor family that has a greater inhibitory effect on CYP3A4 and fewer side effects compared to LPV/r; therefore, ATV/r is easier to tolerate for patients. Moreover, LPV/r has been shown to have better pulmonary permeability [17]. COVID-19 causes a widespread inflammatory response in the body through the cytokine storm mediated by interleukin-6. Some symptoms of COVID-19 stem from this inflammatory reaction. Evidence showed that some protease inhibitors such as atazanavir can reduce symptoms and disease severity to some extent by decreasing the release of interleukin-6 from primary monocytes [18, 19].

Protease inhibitor drugs cause a number of side effects. This includes common side effects of LPV/r as nausea, vomiting, diarrhea, hepatotoxicity, prolonged Q-T on electrocardiogram (ECG), skin rash, hyperlipidemia, and abdominal pain. Furthermore, ATV/r can cause some side effects such as hyperbilirubinemia, rash, fever, nausea, vomiting, diarrhea, high cholesterol, and cough. What is more, hyperbilirubinemia is the most frequent adverse effect of ATV. ATV can cause a reversible, dose-dependent increase predominantly of unconjugated bilirubin [20].

In previous studies, hydroxychloroquine caused some complications such as hemolysis in patients with glucose-6-phosphate dehydrogenase (G6PD) deficiency, bone marrow suppression, cardiomyopathy, some abnormalities in ECG including prolonged PR, QRS, QT interval, and some new-onset cardiac arrhythmias such as bundle branching block (BBB) and atrioventricular block [21, 22]. On the other hand, the definite treatment of COVID-19 is not exactly known and the role and effectiveness of combination therapy in this disease are still a matter to be investigated.

In the present study, we aimed to compare the efficacy and side effects of two combination therapies including atazanavir/ritonavir (ATV/r) or lopinavir/ritonavir (LPV/r) plus hydroxychloroquine among COVID-19 patients.

## 2. Methods

### 2.1. Study Design and Participants

The study was designed as a clinical trial in which the patients who presented moderate or severe COVID-19 disease were recruited based on the diagnosis of the infectious disease specialist. Overall, 108 eligible patients with COVID-19 who were admitted to Ayatollah Khansari Hospital and Amir Al-Momenin Hospital in Arak city were investigated from May to October 2020.

Patients were divided into two groups (*n* = 54). Inclusion criteria were patients with moderate to severe COVID-19 that were hospitalized in the infectious and COVID wards of the hospital besides signing the informed consent. Exclusion criteria were the critical type of the disease that required intensive care unit (ICU) admission and intubation, a mild type of the disease without need to hospitalization, and unwillingness to participate in this study. Moreover, discharge was based on all of the following: (1) improvement in clinical signs and symptoms based on physician's opinion, (2) afebrile status for 72 h without antipyretics, and (3) saturation of peripheral oxygen (SpO2) more than 93% in ambient air without supplemental oxygen.

In the present study, moderate and severe COVID-19 were defined according to WHO guidelines. Therefore, moderate form of COVID-19 is defined as the existence of primary symptoms of pneumonia (dyspnea, cough, and fever) and SpO2 ≥ 90% in ambient air and severe form is defined as profound pneumonia and SpO2 < 90%. The diagnosis approach was based on the WHO guideline so that positive reverse transcriptase-polymerase chain reaction (RT-PCR) of nasopharyngeal and oropharyngeal secretions or clinical manifestations plus findings of chest CT scan was highly suggestive for COVID-19 [7, 13].

This study was approved by the Ethics Committee of Arak University of Medical Sciences (approval ID: IR-ARAKMU.REC.1399.006). The study protocol was also registered as IRCT20200517047485N1. Written informed consents were obtained from the patients or one of the first-degree family members if the patient was unconscious.

### 2.2. Therapeutic Regimens

Patients of one group received hydroxychloroquine tablet 400 mg single dose on the first day and LPV/r (200 mg/50 mg tablet) 2 tablets every 12 hours from the second day for at least 5-7 days.

The other group received hydroxychloroquine 200 mg every 12 hours plus ATV/r 1 tablet (300 mg/100 mg) daily for at least 5-7days. According to physician's judgment, the treatment period was longer whenever needed. The duration of treatment was at least 7 days. In addition, more treatments were also recorded such as other antiviral drugs, corticosteroids, intravenous immunoglobulin (IVIG), vitamin C, antibiotics, analgesic agents, antinausea and vomiting agents, cardiovascular drugs, deep vein thrombosis (DVT), and stress ulcer prophylaxis.

### 2.3. Laboratory Tests

Demographic data, clinical presentations, underlying diseases, drug history, and laboratory and medical data were collected and documented. Clinical symptoms such as fever, sore throat, dyspnea, abdominal pain, and clinical signs including SpO2, pulse rate, respiratory rate, blood pressure, and temperature were recorded at the time of hospital admission. Vital signs and laboratory tests such as white blood cells (WBC), serum electrolytes, liver and kidney enzymes, inflammatory biomarkers consisting of C-reactive protein (CRP), and erythrocyte sedimentation rate (ESR) were extracted from the hospital information system (HIS) and patient's file every day and recorded in questionnaire.

During the hospitalization, the patients were examined daily for the presence or absence of signs and symptoms of COVID-19 disease as well as vital signs and daily laboratory tests which were collected through a questionnaire. The patient's ECG was taken daily or every other day upon admission and during the hospitalization. They were then interpreted by a cardiologist for the presence or absence of arrhythmia who was not aware of the patient's medication.

### 2.4. Statistical Analysis

The statistical analysis was performed using SPSS version 23.0. A *p* value less than 0.05 is considered statistically significant. Descriptive data were calculated as frequency, frequency percentage, and analytical statistics through the chi-square test.

## 3. Results

This study was performed on 108 patients with COVID-19 who were in the two groups of 54 patients. 47.2% of patients were male and 52.8% were female. The majority of patients aged from 60 to 79 years (42.6%). The most common symptoms in both groups were dry cough (67.6%), myalgia (65%), and dyspnea (60.2%), and the least common symptom was skin rash (8.3%).

On admission, 27.8% of patients had SpO2 > 94%, and 41.7% of them had SpO2 between 90 and 94% and 30.6% had SpO2 less than 90%. There was no significant difference between the two groups for the findings of physical examination at the time of admission including fever, blood pressure, respiratory rate, SpO2, and heart rate.

Based on the findings of physical examination and evaluation of patients during the treatment period (Table 1), the number of days with fever (*p* = 1.00), number of days with hypoxia (*p* = 0.343), number of days with cough (*p* = 0.334) and myalgia (*p* = 0.412), and also the length of stay (*p* = 0.479) were not significantly different between the two groups.

Other findings include the number of days with dyspnea (*p* = 0.417), number of days with headache (*p* = 0.647), anorexia (*p* = 0.90), nausea and vomiting (*p* = 1.00), and diarrhea (*p* = 0.315).

Furthermore, sore throat (*p* = 1.00), abdominal pain (*p* = 0.69), and skin lesions (*p* = 0.08) assessed daily for all patients in the course of treatment were not significantly different between the two groups.

On the first day of hospitalization, laboratory tests such as complete blood count (CBC), ESR, CRP, liver enzymes, bilirubin, lipid profile, serum lactate dehydrogenase (LDH) and creatinine phosphokinase (CPK), and international normalized ratio (INR) were checked and were repeated during hospitalization according to the physician's opinion and existing instructions.

In addition, as each treatment regimen can lead to changes in these tests, the trend of changes was categorized as unchanged, ascending, and descending according to the patient's baseline test and the normal range for these tests (Table 2).

Laboratory test findings did not show any significant differences between two groups associated to values of white blood cell (WBC) (*p* = 0.127), hemoglobin (*p* = 0.554), platelets (*p* = 0.906), and lymphocyte percentage (*p* = 0.478) (Table 2). What is more, there was no significant difference for trend of ESR, CRP, creatinine, and liver enzymes (Table 2).

As a highlighted outcome, bilirubin was significantly higher in the group of patients who received ATV/r than the group of patients who received LPV/r (*p* < 0.001). Hyperbilirubinemia is one of the most common side effects of ATV/r, and it is less common for LPV/r.

The other laboratory findings such as lipid profile, blood sugar, CPK, LDH, and INR were not significantly different.

The other significant result was a higher rate of arrhythmia in the LPV/r group than the ATV/r group (*p* = 0.019) (Table 3). In this study, 44 patients suffered arrhythmias during treatment period including 28 patients of the LPV/r group and 16 patients of the ATV/r group. The types of arrhythmias for these patients were sinus bradycardia (*n* = 10), prolonged Q-T (*n* = 9), sinus tachycardia (*n* = 7), first-degree block (*n* = 6), left bundle branch block (RBBB) (*n* = 6), right bundle branch block (RBBB) (*n* = 3), premature ventricular contraction (PVC) (*n* = 2), and premature atrial contraction (PAC) (*n* = 1). These arrhythmias were diagnosed by a cardiologist by comparing the initial ECG in admission day ECG of hospitalization days.

The treatment outcomes were classified as discharge with a good and stable general condition, evidence of complications during the hospital stay that continues until discharge or mortality. There was no significant difference in treatment outcomes between the two groups.

Unfortunately, five investigated patients including 4 females and 1 male (aged from 60 to 80) expired, from whom three patients belonged to the LPV/r group and the two were in the ATV/r group. All these patients had SpO2 less than 90% on admission. Moreover, one had no history of specific underlying disease whereas two had a history of diabetes and hypertension, one with a history of hypertension and coronary heart disease, and the last one with only a history of diabetes.

Our findings indicated that ten patients of the LPV/r group had complications during hospitalization and discharge. In addition, four patients had no increase in SpO2 and were dependent on oxygen on discharge, two patients had DVT, one had pulmonary thromboembolism (PTE), two had pressure ulcer, and one patient developed hemoptysis.

Finally, among the ATV/r group, even patients developed complications during hospitalization and on discharge. Three patients did not have increased SpO2 during hospitalization and on discharge, one patient developed pancreatitis, another had hemoptysis, and one showed gastrointestinal bleeding (GIB).

## 4. Discussion

To the best of our knowledge, in a few studies, outcomes and complications of combination therapy were compared among COVID-19 hospitalized patients. In our clinical trial, the efficacy and side effects of two combination therapies with LPV/r and ATV/r plus hydroxychloroquine were compared between the two groups of COVID-19 patients.

The results of this study indicated that combination therapy with these drugs was not significantly different between two groups in terms of hospitalization length, complications, mortality rate following treatment period, time of alleviation from clinical symptoms, and clinical outcomes.

The rate of hyperbilirubinemia was significantly higher in the ATV/r group than the LPV/r group. In this study, 85.4% of patients that received ATV/r had hyperbilirubinemia. In line with our study, a meta-analysis on the clinical benefit of ATV/r- and LPV/r-based cART in HIV patients demonstrated that the risk of ATV/r-induced hyperbilirubinemia is very high in comparison with LPV/r [16].

Atazanavir-induced hyperbilirubinemia is indirect hyperbilirubinemia that is created by inhibition of the UDP-glucuronyltransferase (UGT) enzyme that conjugates bilirubin, which is associated with genetic characteristics [23]. Moreover, it has been previously reported that UGT1A1∗28 polymorphism increased the risk of atazanavir-induced hyperbilirubinemia in HIV patients [16]. Therefore, the use of pharmacogenetics tests can help to determine a patient's susceptibility to drug toxicity and to facilitate the selection of appropriate medicine.

The results of some studies revealed that boosted atazanavir with ritonavir increases the risk of indirect hyperbilirubinemia compared to atazanavir alone [24, 25].

In a study by Malan et al. which was performed on 400 patients, the rate of hyperbilirubinemia was 59% in the ATV/r group and 20% in the atazanavir group [24].

Interestingly, Moyle G et al.'s study revealed a meaningful decrease in unconjugated and total bilirubin following zinc sulfate (ZnSO_4_) intake and a limited decrease in ATV/r plasma exposure suggesting ZnSO_4_ supplementation may represent a useful medicine in the short-term management of ATV-related hyperbilirubinemia in HIV-infected patients [16]. Therefore, further studies are needed to evaluate the beneficial effects of some supplements in combination with ATV/r to decrease its side effect.

The cardiac arrhythmias rate during the treatment period was significantly higher in the LPV/r group compared to the ATV/r group. In this study, almost all the patients received antibiotic therapy in addition to antiviral therapy including fluoroquinolones and macrolides which may cause arrhythmias. On the other hand, the interaction of LPV/r with these antibiotics, as well as with hydroxychloroquine, which has been used as part of combination therapy against COVID-19 in these patients, might be the cause of higher arrhythmias in the LPV/r group.

Anson et al. showed that LPV/r could predispose patients to arrhythmias such as prolonged Q-T and torsade de point by inhibiting of human ether-a-go-go-related gene (HERG) potassium channels and potassium current (IKr) channels [16]. In Bessière et al.'s study, combination therapy with hydroxychloroquine and azithromycin caused prolonged Q-T in 10.7-36% of patients [16].

Russo et al.'s findings indicated that 23% of patients developed arrhythmias following the COVID-19 treatment by hydroxychloroquine and azithromycin [26].

## 5. Conclusions

In this study, two different combination therapies (ATV/r and LPV/r group) in terms of efficacy and side effects were comparatively investigated. The findings indicated a significant increase in bilirubin in ATV/r receivers compared to LPV/r-treated patients. Arrhythmias were also significantly increased in the LPV/r group compared to the ATV/r group during the treatment. Apart from the rate of hyperbilirubinemia and cardiac arrhythmia, other indices were not statistically various between the two groups. There was no significant difference for mortality rate and complications of treatment regimen between two groups. It seems that the current applying agents are not preferably acceptable to overcome COVID-19 infection, and therefore, more therapeutic antiviral regimens must be studied in this era.

## Figures and Tables

**Table 1:** Comparison of clinical presentations in COVID-19 patients on two different combination therapies.

	Duration (days)	Lopinavir/ritonavir *N* (%)	Atazanavir/ritonavir *N* (%)	*p* value
Febrile	0-3	53 (50)	53 (50)	1.00
	3-5	1 (50)	1 (50)	
Admission	3-5	0 (0)	1 (100)	0.479
	5-7	6 (42.9)	8 (57.1)	
	7-9	23 (53.5)	20 (46.5)	
	9-11	21 (56.8)	16 (43.2)	
	11-13	4 (33.3)	8 (66.7)	
	>14	0 (0)	1 (100)	
Hypoxic	0-3	23 (43.4)	30 (56.6)	0.343
	3-5	18 (62.1)	11 (37.9)	
	5-7	1 (20)	4 (80)	
	7-9	1 (33.3)	2 (66.7)	
	9-11	8 (61.5)	5 (38.5)	
	11-13	3 (60)	2 (40)	
Myalgia	0-5	48 (52.2)	44 (47.8)	0.412
	5-10	6 (40)	9 (60)	
	>10	0 (0)	1 (100)	
Cough	0-5	39 (47.6)	43 (52.4)	0.334
	5-10	15 (60)	10 (40)	
	>10	0 (0)	1 (100)	
Dyspnea	0-5	47 (52.8)	42 (47.2)	0.417
	5-10	6 (35.3)	11 (64.7)	
	>10	1 (50)	1 (50)	
Headache	0-5	51 (49.5)	52 (50.5)	0.647
	5-10	3 (60)	2 (40)	
Anorexia	0-5	51 (50.5)	50 (49.5)	0.90
	5-10	2 (40)	3 (60)	
Nausea	0-5	53 (50)	53 (50)	1.00
	5-10	1 (50)	1 (50)	
Diarrhea	0-5	53 (49.5)	54 (50.5)	0.315
	5-10	1 (100)	0 (0)	
Sore throat	0-5	53 (50)	53 (50)	1.00
	5-10	1 (50)	1 (50)	
Abdominal pain	0-5	3 (42.9)	4 (57.1)	0.696
Skin rash	0-5	2 (22.2)	7 (77.8)	0.082

**Table 2:** Comparison of laboratory findings between COVID-19 cases receiving two types of therapeutics.

Features	Trend	Lopinavir/ritonavir *N* (%)	Atazanavir/ritonavir *N* (%)	*p* value
WBC on admission	No change	42 (55.3)	34 (44.7)	0.127
	Increasing	11 (42.3)	15 (57.7)	
	Decreasing	1 (16.7)	5 (83.3)	
Hb	No change	51 (51)	49 (49)	0.554
	Increasing	0 (0)	1 (100)	
	Decreasing	3 (42.9)	4 (51.7)	
Platelet	No change	35 (48.6)	37 (51.4)	0.906
	Increasing	4 (50)	4 (50)	
	Decreasing	15 (53.6)	13 (46.4)	
Lymphocyte count	No change	37 (54.4)	31 (45.6)	0.478
	Increasing	16 (41.2)	26 (57.9)	
	Decreasing	1 (50)	1 (50)	
ESR	No change	19 (46.3)	22 (53.7)	0.836
	Increasing	34 (52.3)	31 (47.7)	
	Decreasing	1 (50)	1 (50)	
CRP	No change	21 (50)	21 (50)	0.587
	Increasing	32 (51.6)	30 (48.4)	
	Decreasing	1 (25)	3 (75)	
Creatinine	No change	42 (50.6)	41 (49.4)	0.82
	Increasing	12 (48)	13 (52)	
AST	No change	36 (49.3)	37 (50.7)	0.978
	Increasing	17 (51.5)	16 (48.5)	
	Decreasing	1 (50)	1 (50)	
ALT	No change	42 (53.8)	36 (46.2)	0.423
	Increasing	11 (40.7)	16 (59.3)	
	Decreasing	1 (33.3)	2 (66.7)	
ALP	No change	47 (48.5)	50 (51.5)	0.579
	Increasing	6 (66.7)	3 (33.3)	
	Decreasing	1 (50)	1 (50)	
Bilirubin	No change	47 (78.3)	13 (21.7)	**<0.001**
	Increasing	7 (14.6)	41 (85.4)	
Lipid profile	No change	38 (47.5)	42 (52.5)	0.380
	Increasing	16 (57.1)	12 (42.9)	
BS	No change	41 (53.2)	36 (46.8)	0.288
	Increasing	13 (41.9)	18 (58.1)	
CPK	No change	36 (49.3)	37 (50.7)	0.790
	Increasing	17 (53.1)	15 (46.9)	
	Decreasing	1 (33.3)	2 (66.7)	
LDH	No change	37 (50.7)	36 (49.3)	0.841
	Increasing	16 (50)	16 (50)	
	Decreasing	1 (33.3)	2 (66.7)	
INR	No change	50 (52.6)	45 (47.4)	0.139
	Increasing	4 (30.8)	9 (69.2)	

ALP: alkaline phosphatase; ALT: alanine amino transaminase; AST: aspartate amino transaminase; BS: blood sugar; CBC: complete blood count; CPK: creatinine phosphokinase; CRP: C-reactive protein; ESR: erythrocyte sedimentation rate; Hb: hemoglobin; INR: international normalized ratio; LDH: lactate dehydrogenase; WBC: white blood cell. Bold *p* value indicates statistical significance.

**Table 3:** The outcome of two different combination therapies among COVID-19 patients.

		Lopinavir/ritonavir *N* (%)	Atazanavir/ritonavir *N* (%)	*p* value
Arrhythmia		28 (63.6)	16 (36.4)	**0.019**
End of treatment	Discharge	41 (47.7)	45 (52.3)	0.633
	Complication	10 (58.8)	7 (41.20)	
	Expired	3 (60)	2 (40)	

Bold *p* value indicates statistical significance.

## Data Availability

The data that support the findings of this study are available from the corresponding author upon reasonable request.

## Abbreviations

ALP: Alkaline phosphatase
ALT: Alanine amino transaminase
AST: Aspartate amino transaminase
BS: Blood sugar
CBC: Complete blood count
CPK: Creatinine phosphokinase
CRP: C-reactive protein
ECG: Electrocardiogram
ESR: Erythrocyte sedimentation rate
GIB: Gastrointestinal bleeding
Hb: Hemoglobin
INR: International normalized ratio
LDH: Lactate dehydrogenase
PAC: Premature atrial contraction
PTE: Pulmonary thromboembolism
RBBB: Right bundle branch block
SpO2: Saturation of peripheral oxygen
WBC: White blood cell.
